# Conformational Heterogeneity of Cyclosporin A in Cyclophilin 18 Binding

**DOI:** 10.1371/journal.pone.0153669

**Published:** 2016-04-15

**Authors:** Weilin Lin, Andres Quintero, Yixin Zhang

**Affiliations:** B CUBE Center for Molecular Bioengineering, Technische Universität Dresden, Arnoldstrasse 18, 01307 Dresden, Germany; Oak Ridge National Laboratory, UNITED STATES

## Abstract

The immunosuppressive drug cyclosporin A (CsA) binds to its receptor protein cyclophilin 18 (Cyp18) in two distinct kinetic phases, while the mechanism remains elusive. Stopped-flow measurements coupled with titration and competition experiments were used to investigate the puzzling two-phase process of CsA and Cyp18 interaction. This study leads to the dissection of different conformational fractions of either direct fast binding or slow binding with rate-limiting conformational inter-conversion and the real-time measurement of *k*_on_ value (8.34 ± 0.22 x10^6^ M^-1^s^-1^) in solution. Furthermore, our study indicates that the structure of CsA during dissociation from the protein possesses a distribution of conformations different from those in solution under equilibrium condition.

## Introduction

The immunosuppressive drug cyclosporin A (CsA) binds to its receptor protein cyclophilin 18 (Cyp18) in two distinct kinetic phases and then undergoes a dramatic conformational change, which allows the Cyp18/CsA complex to inhibit the immunological target protein phosphatase calcineurin (CaN).[1–3] Insights into this gain-of-function biochemical interaction could help us to design natural product-like inhibitory compounds. We have previously reported a structural study of CsA, which led to the first design of CsA analogues that could mimic the drug’s conformation in the protein/ligand complex and Cyp18-independent CaN inhibition.[1, 4] While the Cyp18/CsA complex represents one of the most complex protein/drug interactions, an interesting but puzzling observation is that CsA possesses slow inhibitory kinetics to Cyp18.[5] Largely because of this, the k_on_ and k_off_ values of CsA to Cyp18 in solution have not been reported, though the IC_50_, K_i_ and k_d_ values can be precisely determined.[6] Given that CsA is poorly soluble in water, the NMR structures can only be obtained in organic solvents.[7, 8] Seven out of eleven amino acids are N-methylated, allowing the cyclic peptide to adopt many different conformations in equilibrium in polar solvents without being stabilized in a certain structure of high percentage.[7–10] We have previously shown that the Sar^3^ modification of CsA impairs the β-II’ turn formation, leads to structure similar to that in the CsA/Cyp18 complex.[1] The resulting Sar^3^ modified CsA analogues could not only inhibit CaN in a Cyp18 independent manner, but also bind to Cyp18 with diminished slow phase. We believe that the heterogeneous and dynamic nature of CsA structure underlies its many biological properties. In this work, we investigated the effect of CsA structural heterogeneity on the binding to its receptor protein Cyp18.

## Materials and Methods

### Stopped flow measurement coupled with titration dissecting the different binding phases

Experiments were carried out at room temperature in HEPES buffer (35 mM HEPES, 150 mM NaCl, 1 mM DTT buffer, pH 7.4) using LS 55 Fluorescence Spectrometer (PerkinElmer) equipped with SFA-20 rapid kinetics stopped-flow accessory. The dead time of the stopped flow is less than 8 ms. The intrinsic fluorescence of Cyp18 was measured as described before.[11] Briefly, 1 mL of 1 μM Cyp18 in one syringe was rapidly mixed with a 1 mL solution in a second syringe containing concentrations of CsA from 0.4 to 5 μM using stopped flow method. The intrinsic fluorescence of Cyp18 was recorded.

### Fluorescence kinetic measurement of Cyp18 upon binding to CsA dissolved in different organic solvents (method A) or after incubation in HEPES buffer (method B)

Method A: 10 μl Cyp18 (100 μM in HEPES buffer) was added to 1980 μl HEPES buffer under continuous stirring, then 10 μl CsA (50 μM in DMSO, THF, or 470 mM LiCl/THF) was added to the mixture. Method B: 10 μl CsA (50 μM in DMSO, THF, or 470 mM LiCl/THF) was added to 1980 μl HEPES buffer under continuous stirring and the mixture was incubated for 10 min. Then 10 μl Cyp18 (100 μM in HEPES buffer) was added to the mixture. The intrinsic fluorescence of Cyp18 was recorded.

### Fluorescence kinetic measurement of Cyp18 upon binding to CsA dissolved in different solution with various pH

CsA sample preparation: 5 μL CsA (500 μM in DMSO) was mixed with 45 μL HCl solution of different concentrations (100 mM, 1 mM, 0.01 mM) or NaOH solution of different concentrations (100 mM, 1 mM, 0.01 mM) or HEPES buffer and kept in room temperature for more than 30 mins. For the fluorescence kinetic measurement, 10 μl Cyp18 (100 μM in HEPES buffer) was added to 1980 μl HEPES buffer under continuous stirring, then 10 μl CsA (50 μM in 10% DMSO with different pH) was added to the mixture. The intrinsic fluorescence of Cyp18 was recorded.

### Association rate measurement between CsA and Cyp18 (*k*_on-fast_) using stopped flow method

1 mL 500 nM Cyp18 in one syringe was rapidly mixed with 1 mL solution in a second syringe containing concentrations of CsA from 4 to 12 μM. The intrinsic fluorescence of Cyp18 was recorded. 50% initial concentration of CsA, corresponding to the fast binding conformer, is used to calculate the *k*_on-fast_ value.

### Fluorescence kinetic measurement of Cyp18 upon binding to CsA during its dissociation from Cyp40/CsA complex without (method A) or with (method B) pre-equilibration

To obtain the fluorescence signal shifting of buffer upon the additional of THF and LiCl/THF, 10 μL of THF or LiCl/THF was directly added 1990 μL buffer with or without the addition of Cyp18 under continuous stirring. (S1 Fig) Method A: 1 mL 1 μM Cyp18 in one syringe was rapidly mixed with 1 mL 1 μM Cyp40-CsA complex (pre-incubated for 30 min) using the stopped flow method as described before. The increase of the signal was fitted with first order (y = y_0_+a×e^-kt^). Method B: After the equilibration of a volume of 250 μl NeutrAvidin® Agarose Resin in HEPES buffer, the resin was incubated with 1ml biotin labeled CsA (10 μM in HEPES buffer) [12] for 30 min. The unbound biotin labeled CsA was removed by washing 3 times with HEPES buffer. The Cyp40-CsA complex was formed by incubating CsA and Cyp40 in 2 ml HEPES buffer at final concentration of 0.5 μM for 30min. Then Cyp40 was removed by the incubation of the mixture with the CsA-conjugated resin for 30 min. After spinning down the resin, a volume of 1990 μl supernatant was transferred to the cuvette to measure the protein intrinsic tryptophan fluorescence and then 10 μl Cyp18 (20 μM in HEPES buffer) was added to the cuvette under stirring. The increase of the signal was fitted with first order (y = y_0_+a×e^-kt^).

## Results

The slow inhibition of Cyp18 by CsA was first observed in a time-dependent Cyp18 inhibition assay.[5] A fast phase followed by a slow phase of Cyp18 inhibition was detected when CsA was added to Cyp18. The ratios of two phases were dependent on the solvents used to dissolve CsA. Similar two-phase kinetics has been obtained through measuring the increase of intrinsic fluorescence of Cyp18.[1] Cyp18 contains a single tryptophan (W121) at its active site, while the fluorescence intensity of tryptophan increases upon binding to CsA.

**Fig 1:**
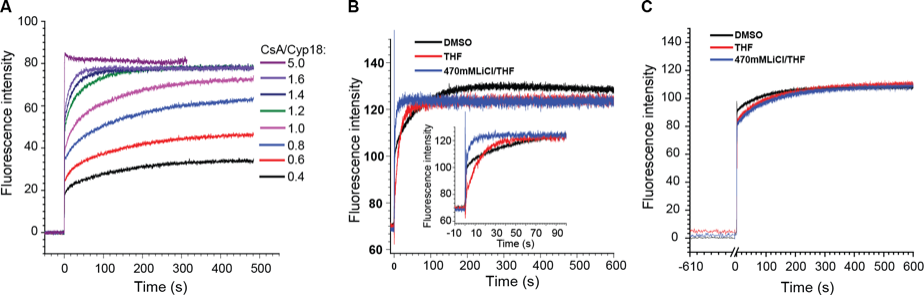
Conformational heterogeneity of CsA in Cyp18 binding. (**A**) Fluorescence intensity time courses of Cyp18 (final concentration of 500 nM) upon the addition of various concentrations of CsA using stopped flow. The graph is plotted after subtraction of the initial fluorescence intensity of free Cyp18. Results shown are the average of two independent experiments. The values shown in the inset represent the different ratios of CsA to Cyp18. (**B**) Fluorescence intensity time courses of Cyp18 upon the addition of CsA dissolved in DMSO (Black), THF (Red) and LiCl/THF (Blue) under continuous stirring. The graph is plotted after subtraction of the reference curve. Results shown are the average of two independent experiments. The inserted graph represents the scale from -10 s to 100 s. (**C**) Fluorescence intensity time course of Cyp18 upon binding to CsA after incubation in HEPES buffer for 10min (black, red and blue curves are CsA dissolved in DMSO, THF and LiCl/THF before adding to HEPES buffer, respectively). The graph is plotted after subtraction of the reference curve. Results shown are the average of two independent experiments.

To understand the reason that causes the two binding phases, we performed a series of titration experiments. As shown in Fig **1A**, when CsA concentrations were equal to or lower than that of Cyp18, the ratios between two phases remained constant (an average of 52.1% of signal increase in the fast phase, as calculated from S**1** Table). Further increasing the CsA concentration reduced the amplitude of the second phase and changed its kinetics. When CsA is in large excess, the second phase could be completely abolished (e.g. CsA concentration is 5-times higher than that of Cyp18). These results suggested the conformational heterogeneity of CsA in Cyp18 binding. When CsA concentration is lower than or equal to that of Cyp18, there is no competition among the different CsA conformers for Cyp18 binding. When CsA concentration is higher than that of Cyp18, the fast binding conformer competes with the slow binders, thus reduces the amplitude of the slow binding phase.

To further demonstrate that these two binding phases are associated with conformational fractions of CsA, we have performed experiments with CsA dissolved in different organic solvents. As shown in Fig 1B, adding CsA dissolved in THF to Cyp18 aqueous solution resulted in significantly increased amplitude of the second binding phase, as compared to those of DMSO, whereas THF/LiCl caused remarkably reduced amplitude of the second binding phase. Moreover, the kinetic courses of second phase differ from each other remarkably. These results are in good agreement with the time-dependent enzyme inhibition assay, as CsA in THF/LiCl and THF possesses stronger and weaker initial inhibition than that in DMSO, respectively[5]. Interestingly, incubation of THF/LiCl or THF dissolved CsA in aqueous solution led to the recovery of the binding profile of CsA dissolved in DMSO (Fig **1C**), excluding the possibility that the solvent effects are caused by changing Cyp18 protein structure by organic solvents. This experiment also demonstrated the reversible effect of various solvents on CsA conformation. We have also performed experiments with CsA dissolved in aqueous solutions of different pH (**S2** Fig). Different from the remarkably effect on binding kinetics caused by various solvents, pH exhibited only minor effects.

**Fig 2:**
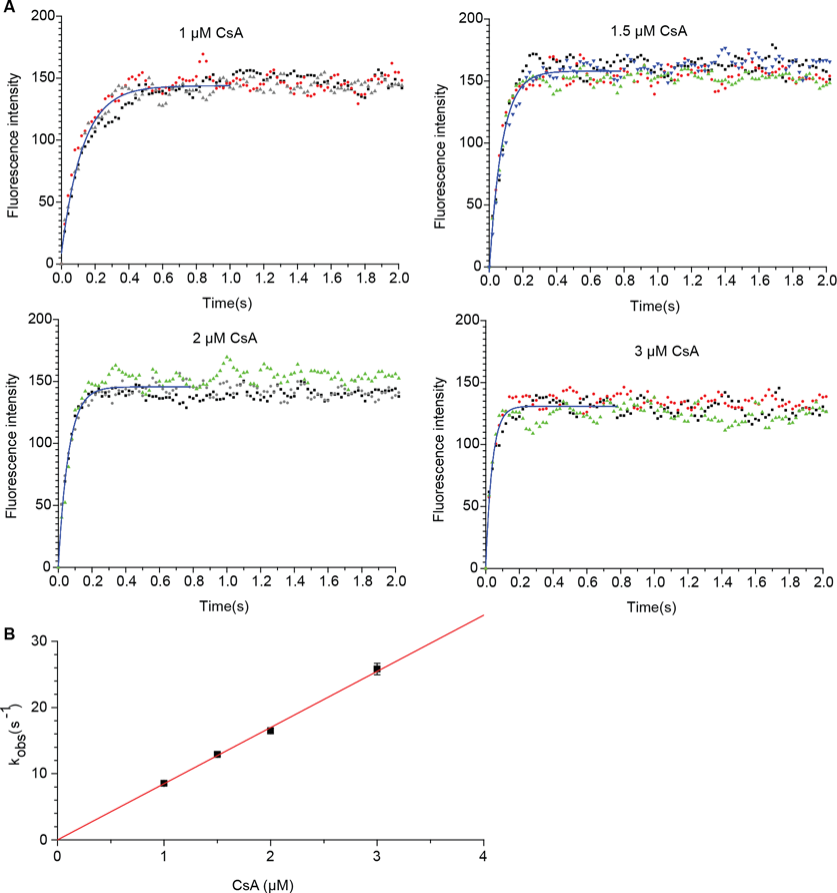
Association rate measurement using stopped flow method. (**A**) Fluorescence intensity time courses of Cyp18 (final concentration of 250 nM) upon the addition of various concentrations of CsA using stopped flow. The concentration of CsA shown in the figure is only the concentration of fast binding conformer (52.1% of the original concentration). The graph is plotted after subtraction of the fluorescence intensity of Cyp18. The raw data from the triplicates are displayed as dots and the first order global fitting as a blue line. (**B**) CsA concentration-dependent, linear increasing of apparent association rate of the fast phase (*k*_obs_). The *k*_on-fast_ value is equated to the slope of the line.

To determine the association rate of the fast phase (k_on-fast_), stopped flow method was performed with CsA in large excess (Fig **2**). This in-solution measurement resulted in a *k*_on-fast_ value of 8.34 ± 0.22 x 10^6^ M^-1^s^-1^, 20-time faster than the association rate determined using surface plasma resonance (SPR) when the Cyp18 was immobilized [13]. This observation was not unexpected, because the on-chip kinetics is composed of both the fast and slow phases. We have recently determined the *k*_off_ value of Cyp18/CsA in solution using a CsA analogue that can quench the Cyp18 intrinsic fluorescence. (unpublished data; Weilin Lin, Frank Erdmann, Andres Quintero, Gunter Fischer, Yixin Zhang) Importantly, the resulting *k*_d_ (8.2 nM) calculated using the *k*_on-fast_ value and the *k*_off_ value (6.81±0.22×10–2 s^-1^) is in good agreement with the *K*_i_ value determined by enzyme inhibition assay and *k*_d_ value determined by isothermal titration calorimetry [6]. This also indicates that the slow phase is not directly associated with the protein-ligand interaction.

**Fig 3:**
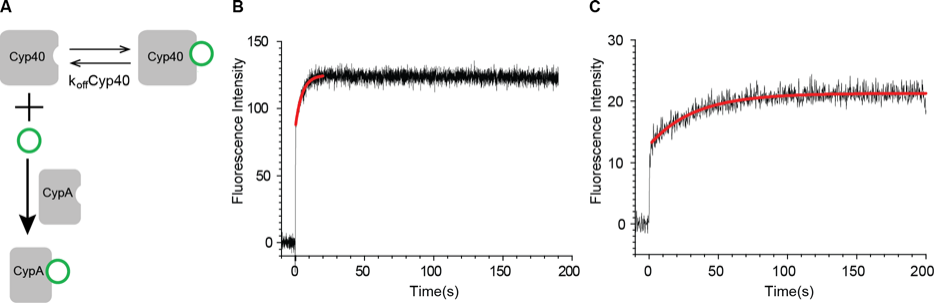
Probing the transient structure of CsA during dissociation from cyclophilin. (**A**) Scheme for measuring the transient structure of CsA during dissociation from Cyp40. (**B**) Fluorescence intensity time course of Cyp18 upon binding to CsA during its dissociation from the Cyp40/CsA complex. The curve was plotted after subtraction of the initial fluorescence intensity of Cyp40. One equivalent of Cyp18 was injected through stopped flow. The time course is fitted to a first-order reaction (*k* = 0.205 s^-1^). (**C**) Fluorescence intensity time course of Cyp18 upon binding to the dissociated CsA from Cyp40. The Cyp40/CsA complex was incubated with a CsA resin to remove the dissociated Cyp40 in solution. After incubating the supernatant at room temperature for half hour, Cyp18 was added and the fluorescent time course was recorded. The k_obs_ value of 0.03067 s^-1^ of the second slow phase is 7 times slower than the single phase kinetics in (B).

To understand the association and dissociation processes between protein and ligand, it is more challenging to analyze the transient structure of ligand when it leaves the binding pocket of the receptor protein. When CsA leaves the binding pocket of cyclophilin, does it adopt the fast binding conformation, or the slow binding conformation, or a mixture of both? We designed an experiment to answer this question (Fig **3A**). The *K*_i_ value between Cyp40 and CsA is 10-times higher than that between Cyp18 and CsA. Cyp40 does not possess the tryptophan at its active site, thus CsA binding does not change the fluorescent signal. We incubated CsA (1.0 μM) and Cyp40 (1.0 μM) at room temperature to form the Cyp40/CsA complex. One equivalent of Cyp18 was then injected through stopped flow, and the change of fluorescent signal was recorded. As shown in Fig **3B**, given that the interaction between CsA and Cyp40 produced no signal change, the resulting kinetics is caused by the binding of CsA to Cyp18, during the dissociation of the ligand from Cyp40. The kinetics is first order (*k* = 0.205 s^-1^), substantially different from the two-phase binding kinetics observed in the direct Cyp18-CsA binding assay and remarkably faster than the slow binding phase. To demonstrate that the dissociated CsA from Cyp40 can re-equilibrate to a mixture of different structures, the Cyp40/CsA complex was incubated with a CsA resin to remove the dissociated Cyp40 in solution. After incubating the supernatant at room temperature for half hour, Cyp18 was added and the two-phase binding kinetics of CsA to Cyp18 was recovered (Fig **3C**). Therefore, we can conclude that most ligands leaving the Cyp40 binding pocket adopt the fast binding conformation, while the observed first order kinetics reflect the k_off_ value of CsA from Cyp40.

## Discussion

For studying protein folding and dynamics, the molecule of interest can be considered as a mixture of structures undergoing conformational changes of different rates [14, 15], whereas the inter-conversion rates for most small organic compounds are relatively fast. CsA is a cyclic undecapeptide, which can be viewed as something between a simple organic compound and a small protein domain. Furthermore, seven of the eleven residues of CsA are N-methylated, which prevents the amide bonds being stabilized in their *trans* conformations. The intrinsic dynamics and conformational heterogeneity of CsA as well as its poor water solubility made it difficult to elucidate the structural details associated with the CsA-Cyp18 binding kinetics in aqueous solution. We speculated the following three models to explain the two binding phases between CsA and Cyp18:

1. Induced-fit model, in which all CsA molecules bind to Cyp18 with a fast kinetics, followed by a slow induced-fit process to reach maximal inhibition. According to this model, the slow enzyme inhibition and fluorescence kinetics are entirely associated with the conformational change induced upon binding to Cyp18.
2. Conformational selection model, in which CsA has multiple conformations and a fraction of them (active conformer) binds to Cyp18 with fast kinetics (k_on-fast_). Other CsA molecules will undergo conformational change and only the active conformer can bind to Cyp18. After the binding, an induced fit process could occur in the protein/ligand complex to reach the final conformation, which is not associated with the observed slow enzyme inhibition and fluorescence kinetics.
3. Parallel binding model, in which the active conformer binds to Cyp18 with fast rate, and the slow binding conformers can bind to Cyp18 either through an induced-fit mechanism or after a conformational change to the active conformer.

The titration experiments have excluded the possibility that the binding is entirely governed through an induced-fit model (Model **I**). By increasing the ratio of CsA to Cyp18, the amplitude of second phase can be diminished. This observation demonstrates the structural heterogeneity of CsA in protein binding. Moreover, changing the initial condition through dissolving CsA in different organic solvents (THF and THF/LiCl) also shows that solvent-induced structural changes will lead to different binding profiles. Different solvents caused changes not only in the amplitudes of second slow binding phase, but also the kinetic courses. This observation indicates that different solvents can induce different conformational fractions of CsA, which bind to Cyp18 with different rates. NMR measurements of CsA in different solvents (e.g. DMSO, chloroform, benzene, and THF/LiCl) have shown that CsA adopts different inter-convertible conformations. [7–10]

The slow binding conformer may need to convert to the active conformer before binding to Cyp18 (Model II). Thus, the rate of slow binding represents the apparent converting rate. When the concentration of CsA is lower than that of Cyp18, the slow binding conformers will bind to Cyp18 once they convert to the active conformation with the rate of k_s-f_ (from slow binding conformer to active conformer). First order global fitting of the slow phases of these curves ([CsA] < [Cyp18]) resulted in the k_s-f_ value of 8.25 ± 0.03 x 10^−3^ s^-1^. (**S3** Fig) As the active conformer is 52.1%, the k_f-s_ (from active conformer to slow binding conformer) value is 7.58 ± 0.03 s^-1^ (k_s-f_ * [slow binding conformer] = k_f-s_ * [active conformer]). Berkeley Modenna method[16] was used to fit the curves including the fast phases at different concentrations (except the ratio of 5:1 (CsA:CypA) when the second phase was abolished). Interestingly, the binding kinetics, independent from the different concentrations of free Cyp18 in solution, could be fitted to an apparent converting rate of 0.00794 S^-1^ (S4 Fig and S2 Table). This result demonstrates that the model II is the most dominant process of CsA to bind to Cyp18, though we cannot exclude the possibility that a small conformational fraction of CsA can still bind to Cyp18 through an induced-fit mechanism (model III).

## Summary

Our work provides the evidence that the two-phase binding kinetics of CsA to Cyp18 is caused by the ligand conformational heterogeneity. This leads to the dissection of different conformational fractions of either direct fast binding or slow binding with rate-limiting conformational inter-conversion. Moreover, the competition experiment described in Fig **3A** allowed us to interrogate the transient structure of a ligand during dissociation from its target protein. The measurement indicated that the structure of CsA during dissociation from Cyp18 possesses a distribution of conformations different from those in solution under equilibrium condition. The methods described in this study could also be applied to investigate other protein-protein or protein-ligand interaction, which involves highly dynamic macromolecule that does not possess a stably folded structure.

## Supporting Information

S1 Fig **Time courses of Cyp18 intrinsic fluorescence intensity upon to the adding of THF(A) or LiCl/THF(B).** (A) Time courses of Cyp18 intrinsic fluorescence with (Red) or without (Black) subtraction of reference curve (only THF). The arrows show the procedures of the experiments and the curves before and after subtraction of reference curve. (B) Time courses of Cyp18 intrinsic fluorescence with (Red) or without (Black) subtraction of reference curve (only LiCl/THF). The arrows show the procedures of the experiments and the curves before and after subtraction of reference curve.
(PDF)

S2 Fig Fluorescence intensity time courses of Cyp18 upon the addition of CsA dissolved in different pH under continuous stirring. (PDF)

S3 Fig First order global fitting when CsA was not more than CypA. The values shown in the inset represent the different ratios of CsA to CypA. The k value was 8.25 ± 0.03 x 10^−3^ s^-1^.
(PDF)

S4 Fig Time courses of CsA/Cyp18 complex forming. Black curves are plotted by the data converted from Fig **1A** (80.87 of fluorescence intensity was equaled to 500 nM CypA/CsA complex) and the blue curves are the global fitting curves using Berkeley modenna. The final parameters after fitting were shown in S2 Table (down numbers).
(PDF)

S1 Table Fluorescence intensity of Cyp18 upon the binding of CsA. (PDF)

S2 Table The parameters used to do the fitting by Berkeley modenna (Up numbers) and the parameters observed after the fitting (Down number). The range of k_s-f_ and k_f-s_ used for fitting was board (highlight), whereas the fitting showed closed value to the predicted, this indicated the conformational selection model was the most dominant process of CsA binding to Cyp18 in aqueous solution.
(PDF)
