# Supplementary material for: Conformational Heterogeneity of Cyclosporin A in Cyclophilin 18 Binding
Source: PLoS One. 2016 Apr 15;11(4):e0153669. doi: 10.1371/journal.pone.0153669 (PMC4833397; doi:10.1371/journal.pone.0153669)
Supplement: S1 Fig — Time courses of Cyp18 intrinsic fluorescence intensity upon to the adding of THF(A) or LiCl/THF(B). (A) Time courses of Cyp18 intrinsic fluorescence with (Red) or without (Black) subtraction of reference curve (only THF). The arrows show the procedures of the experiments and the curves before and after subtraction of reference curve. (B) Time courses of Cyp18 intrinsic fluorescence with (Red) or without (Black) subtraction of reference curve (only LiCl/THF). The arrows show the procedures of the experiments and the curves before and after subtraction of reference curve. (PDF) [file pone.0153669.s001.pdf]

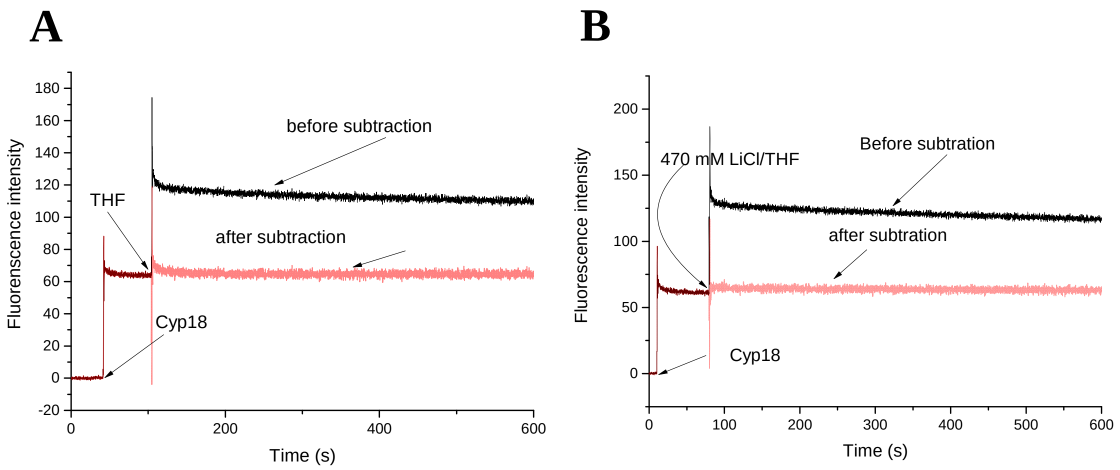

S1 Fig. Time courses of Cyp18 intrinsic fluorescence intensity upon to the adding of THF(A) or LiCl/THF(B). (**A**) Time courses of Cyp18 intrinsic fluorescence with (Red) or without (Black) subtraction of reference curve (only THF). The arrows show the procedures of the experiments and the curves before and after subtraction of reference curve. (**B**) Time courses of Cyp18 intrinsic fluorescence with (Red) or without (Black) subtraction of reference curve (only LiCl/THF). The arrows show the procedures of the experiments and the curves before and after subtraction of reference curve.
