# Supplementary material for: Conformational Heterogeneity of Cyclosporin A in Cyclophilin 18 Binding
Source: PLoS One. 2016 Apr 15;11(4):e0153669. doi: 10.1371/journal.pone.0153669 (PMC4833397; doi:10.1371/journal.pone.0153669)
Supplement: S2 Fig — (PDF) [file pone.0153669.s002.pdf]

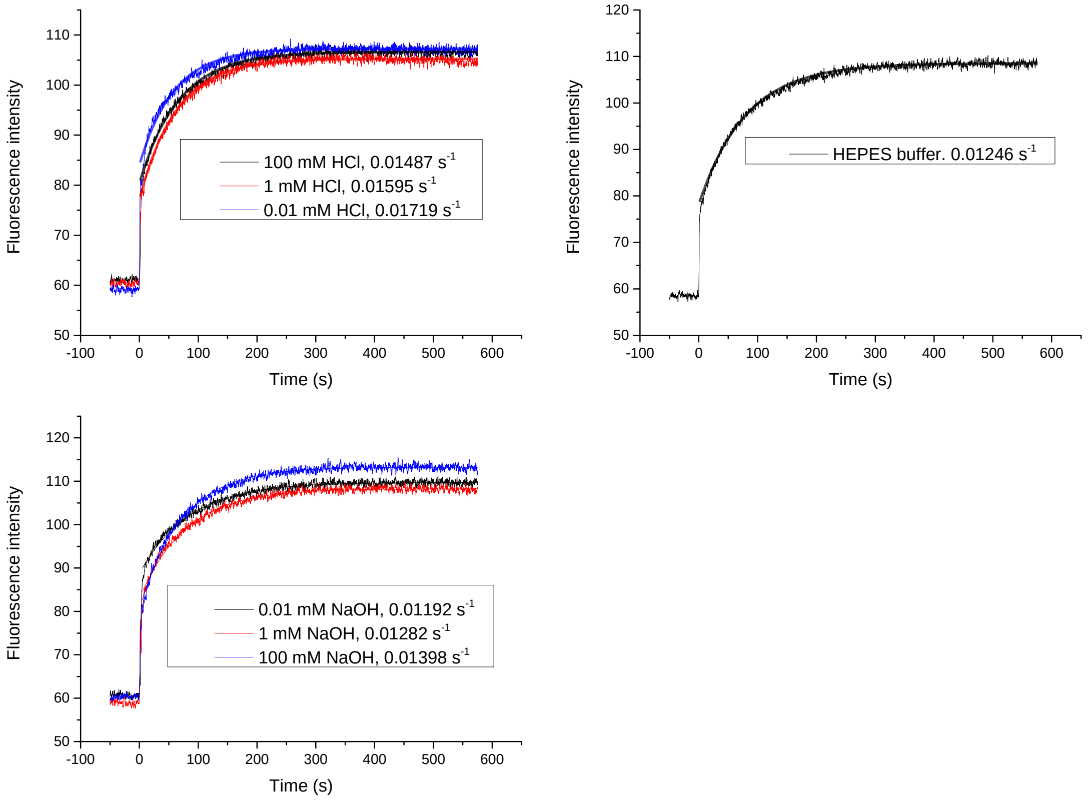

S2 Fig. Fluorescence intensity time courses of Cyp18 upon the addition of CsA dissolved in different pH under continuous stirring.
