# Supplementary material for: Conformational Heterogeneity of Cyclosporin A in Cyclophilin 18 Binding
Source: PLoS One. 2016 Apr 15;11(4):e0153669. doi: 10.1371/journal.pone.0153669 (PMC4833397; doi:10.1371/journal.pone.0153669)
Supplement: S3 Fig — The values shown in the inset represent the different ratios of CsA to CypA. The k value was 8.25 ± 0.03 x 10−3 s-1. (PDF) [file pone.0153669.s003.pdf]

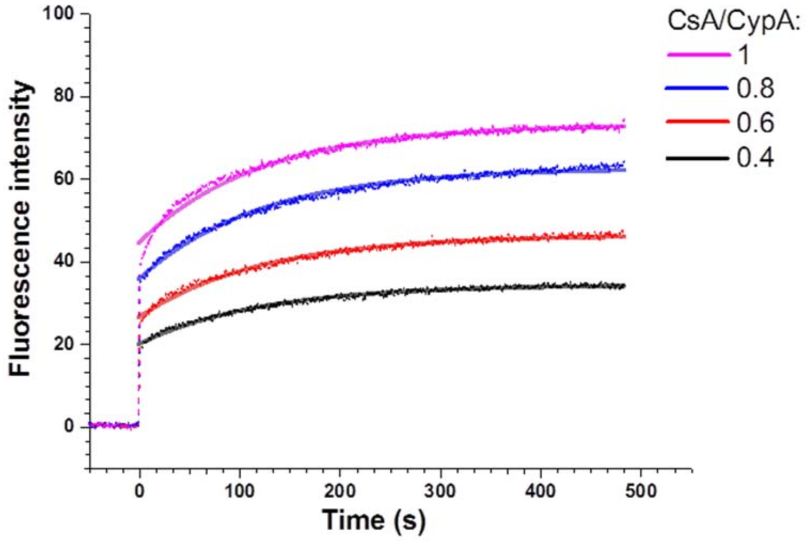

S3 Fig. First order global fitting when CsA was not more than CypA. The values shown in the inset represent the different ratios of CsA to CypA. The k value was 8.25 ± 0.03 s^{-1}.
