# Supplementary material for: Conformational Heterogeneity of Cyclosporin A in Cyclophilin 18 Binding
Source: PLoS One. 2016 Apr 15;11(4):e0153669. doi: 10.1371/journal.pone.0153669 (PMC4833397; doi:10.1371/journal.pone.0153669)
Supplement: S4 Fig — Black curves are plotted by the data converted from Fig 1A (80.87 of fluorescence intensity was equaled to 500 nM CypA/CsA complex) and the blue curves are the global fitting curves using Berkeley modenna. The final parameters after fitting were shown in S2 Table (down numbers). (PDF) [file pone.0153669.s004.pdf]

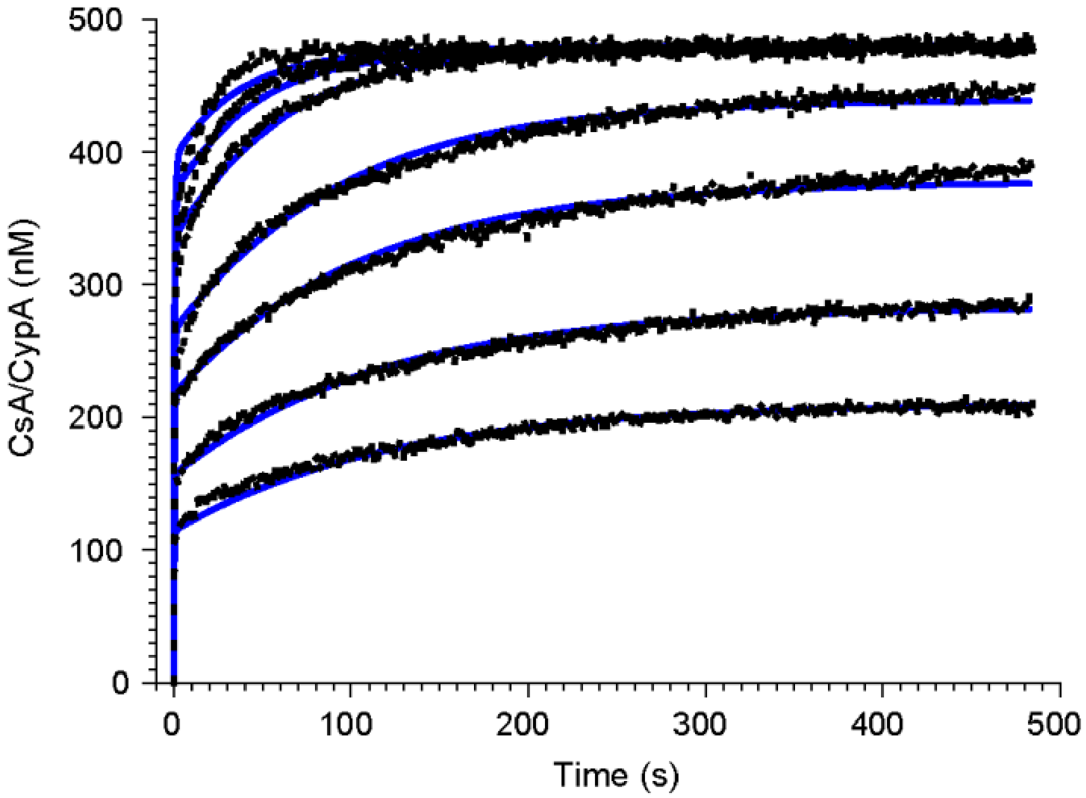

S4 Fig. Time courses of CsA/Cyp18 complex forming. Black curves are plotted by the data converted from Fig **1A** (80.87 of fluorescence intensity was equaled to 500 nM CypA/CsA complex) and the blue curves are the global fitting curves using Berkeley modenna. The final parameters after fitting were shown in S2 Table (down numbers)
