# Supplementary material for: Conformational Heterogeneity of Cyclosporin A in Cyclophilin 18 Binding
Source: PLoS One. 2016 Apr 15;11(4):e0153669. doi: 10.1371/journal.pone.0153669 (PMC4833397; doi:10.1371/journal.pone.0153669)
Supplement: S1 Table — (PDF) [file pone.0153669.s005.pdf]

S1 Table. Fluorescence intensity of Cyp18 upon the binding of CsA.

CsA/Cyp18	Fast increasement	Total increasement	Relative fast increasement (%)	
5.0	83.30	80.87	103.00	
1.6	53.26	77.96	68.32	
1.4	49.75	77.78	63.96	
1.2	47.82	78.23	61.13	
1.0	36.98	72.47	51.03	Average 52.10
0.8	34.20	62.92	54.36	
0.6	23.32	46.20	50.47	
0.4	17.68	33.66	52.53	
