# Supplementary material for: Conformational Heterogeneity of Cyclosporin A in Cyclophilin 18 Binding
Source: PLoS One. 2016 Apr 15;11(4):e0153669. doi: 10.1371/journal.pone.0153669 (PMC4833397; doi:10.1371/journal.pone.0153669)
Supplement: S2 Table — The range of ks-f and kf-s used for fitting was board (highlight), whereas the fitting showed closed value to the predicted, this indicated the conformational selection model was the most dominant process of CsA binding to Cyp18 in aqueous solution. (PDF) [file pone.0153669.s006.pdf]

S2 Table. The parameters used to do the fitting by Berkeley modenna (Up numbers) and the parameters observed after the fitting (Down number). The range of $k_{s\text{-}f}$ and $k_{f\text{-}s}$ used for fitting was board (Table 1, highlight), whereas the fitting showed closed value to the predicted, this indicated the conformational selection model was the most dominant process of CsA binding to Cyp18 in aqueous solution.

CsA/Cyp18	Cyp18 (nM)[a]	CsA (nM)[a]	$k_{on\text{-}fast}(nM^{-1}s^{-1})$[b]	$koff(s^{-1})$[b]	$k_{f\text{-}s}(s^{-1})$[c]	$k_{s\text{-}f}(s^{-1})$[c]
1.6	450 ~ 550<br>503.74	344.88 ~ 421.52<br>383.91				
1.4	450 ~ 550<br>507.77	301.77 ~ 368.83<br>352.22				
1.2	450 ~ 550<br>520.11	258.66 ~ 316.44<br>315.00				
1.0	450 ~ 550<br>525.00	215.55 ~ 263.45<br>245.00	0.00792-0.00876<br>0.00833	0.0647-0.0715<br>0.0700	**0.000825-0.0825<br>0.00794**	**0.000758-0.0758<br>0.00709**
0.8	450 ~ 550<br>503.30	172.44 ~ 210.76<br>200.91				
0.6	450 ~ 550<br>492.59	129.33 ~ 158.07<br>143.91				
0.4	450 ~ 550<br>504.58	86.22 ~ 105.38<br>104.78				

a. For Cyp18, the concentration range: 500 nM X 0.9 ~ 500 nM X 1.1.
   For CsA, the concentration range: 500 nM X ratio X 0.479 X 0.9 ~ 500 nM X ratio X 0.479 X 1.1
b. The range: the obtained value X 0.95 ~ the obtained value X 1.05
c. The range: the obtained value X 0.1 ~ the obtained value X 10
